# The Effect of B Coating in Enhancing Properties of Al/Diamond Composites

**DOI:** 10.3390/ma18092117

**Published:** 2025-05-05

**Authors:** Jiaxiong Li, Muqing Hou, Haiyuan Chen, Huan Yu, Jixue Zhou, Jianhua Wu, Xitao Wang

**Affiliations:** Advanced Materials Institute, Qilu University of Technology (Shandong Academy of Sciences), Jinan 250014, China; 15536307728@163.com (J.L.);

**Keywords:** composites, B coating, interfacial structure, thermal-physical properties

## Abstract

Al/diamond composites are considered a new generation of potential thermal management materials. In this study, the uniform B coating on the surface of diamond particles was prepared by the vacuum thermal diffusion method. Subsequently, Al matrix composites reinforced with B-coated diamond particles were produced by the gas pressure infiltration method. The plating time was varied from 0 to 120 min to investigate the effect of B coating on the thermal conductivity and thermal expansion of the Al/B-diamond composites. The thermal conductivity for the Al/B-diamond composites increased from 422 W/m·K to 562 W/m·K with prolonged plating time from 0 to 90 min. Correspondingly, the coefficient of thermal expansion was tailored from 7.0 × 10^−6^/K to 5.8 × 10^−6^/K. The enhanced thermal conductivity and decreased coefficient of thermal expansion were attributed to strong interfacial bonding in Al/B-diamond composites. The results indicate that the appropriate thickness of B coating is a viable method to improve interfacial bonding between the Al matrix and B-coated diamond particles.

## 1. Introduction

With the development of advanced high-power density electronic components, which brings a rapid increase of heat per unit volume. The heat flux in very large scale integration (VLSI) reached up to 10^3^–10^4^ W/cm^2^ [1]. The traditional packaging materials with low thermal conductivity, such as Al/SiC composite (250 W/m·K) [2], are no longer adequate for meeting the heat sinks. The heat dissipation problem of electronic devices has become a crucial bottleneck restricting their development. There is an urgent need to develop high-performance electronic packaging materials that can dissipate heat rapidly [1,3]. Diamond has high thermal conductivity (up to 2000 W/m·K) and a low coefficient of thermal expansion (1 × 10^−6^/K). Hence, diamond particle-reinforced aluminum matrix (Al/diamond) composites are a highly promising thermal management material owing to the high thermal conductivity, coefficient of thermal expansion that matches with semiconductor materials, low density, etc. [4,5,6]. However, the interface phase of Al_4_C_3_ is often formed in the preparation process of the Al/diamond composites [7]. As is well known, the hygroscopic behavior of Al_4_C_3_ has been observed in humid conditions, which will induce the interfacial debonding and undermine the properties of the Al/diamond composites [8,9]. To avoid the above problems, an effective method is to modify the surface of diamond particles using carbide-forming elements such as Si [10,11,12], W [13,14,15], Mo [16,17,18], and Ti [19,20,21] et, and metal elements such as Ni [22]. The formed carbide layer has three main functions. Firstly, it improves the wettability between the Al matrix and diamond particles, enhancing the interface bonding of the Al/diamond composite. Secondly, it prevents the formation of Al_4_C_3_. Finally, it can reduce the phonon mismatch between Al and diamond particles, reducing the interface thermal resistance.

B coating on the surface of diamond particles can improve the properties of diamond particles reinforced metal matrix composites. Mudholkar et al. [23] obtained high wear-resistant composites by autocatalytic Ni-B coating on the surface of diamond particles. Simultaneously, the B element can react with C atoms on the surface of diamond to form B_4_C. Ras et al. [24] obtained the B_4_C-coated diamond particles by heating a powder mixture of diamond particles, H_3_BO_3_, and amorphous B at 1150 °C under an argon atmosphere for dwell times of 2–6 h. Compared with other carbides, B_4_C has low density (2.52 g/cm^3^), appropriate thermal conductivity (67 W/m·K), and high phonon velocity, which is between metal matrix and diamond [25,26]. This can effectively reduce the phonon mismatch between metal and diamond particles, improving the thermal conductivity of diamond particles reinforced metal matrix composites. Bai et al. [27] employed B into the Cu matrix to form B_4_C on the diamond surface, improving the interfacial bonding and the thermal conductivity of the Cu-B/diamond composites. Hu et al. [28] prepared Cu/B-diamond composites by the vacuum hot pressure method with a thermal conductivity of 665 W/m·K. The above results further confirm the beneficial effects of B_4_C on the thermal conductivity of the composites. At the same time, B_4_C has stable chemical properties and good wettability with Al [29]. Hence, the B_4_C coating on the surface of diamond particles is a potentially effective transition layer for improving the interfacial bonding and avoiding the reaction between the Al matrix and diamond to form Al_4_C_3_. Sun et al. [30] found that the B_4_C coating on diamond significantly enhances the bending strength and thermal conductivity of the Al/diamond composites. The highest thermal conductivity of 352.7 W/m·K was achieved in the composite with 50 vol.% B-coated diamond particles, which was prepared by the powder metallurgy method. However, the influence of the B coating on the interfacial microstructure of the Al/B-diamond composites has not been thoroughly analyzed.

In this work, the B coating on diamond particles is achieved by the vacuum thermal diffusion method. Al matrix composites reinforced with B-coated diamond particles (Al/B-diamond composites) are subsequently fabricated through the gas pressure infiltration method. The effect of B coating on the interfacial microstructure and thermophysical properties of Al/B-diamond composites is investigated. The thermal conductivity, coefficient of thermal expansion of the composites are measured and discussed in relation to the interfacial microstructure.

## 2. Materials and Methods

### 2.1. Materials

Commercial Al (purity 99.99 wt.%, Aluminum Corporation of China, Beijing, China) was used as the metal matrix. HHD600-type synthetic diamond particles (particle size 150–180 μm, Henan Huanghe Whirlwind Co., Zhengzhou, Henan, China) were employed as reinforcement. Amorphous B powders (purity 99 wt.%, particle size ~20 μm, Aladdin, Shanghai, China) were utilized as the B source during the coating process.

### 2.2. Preparation of Al/Diamond Composites

**Figure 1:**
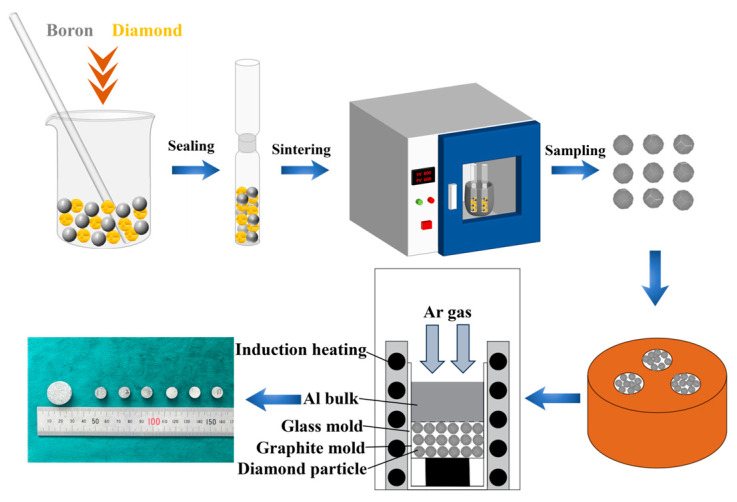
The schematic diagram of the preparation process of Al/B-diamond composites.

Diamond particles and B powders were homogeneously mixed with the mass ratio of 10:1, then subjected to isothermal treatment at 1250 °C for 30–120 min under 10^−1^ Pa to achieve B-coated diamond particles. The B-coated diamond particles were densely packed in a graphite mold with the Al bulk on the top. Then the mold was placed in the gas infiltration furnace. The furnace was heated to 800 °C with a rate of 50 °C/min under 10^−3^ Pa. Afterwards, the high-purity Ar gas was used to infiltrate molten Al into the gaps between diamond particles at 1 MPa for 30 min. Finally, the samples were cooled to room temperature in the furnace. The schematic diagram of the preparation process of the Al/diamond composites is shown in Figure 1.

### 2.3. Characterization and Testing

The morphology, phase structure, and bonding behavior of the B-coated diamond particles were investigated by scanning electron microscope (SEM, ZEISS EVOMA 10, Carl Zeiss AG, Oberkochen, BW, Germany), X-ray diffraction (XRD, Bruker D8 Advance, Berlin, Germany), and X-ray photoelectron spectroscopy (XPS, ESCALAB 250Xi, Waltham, MA, USA). The microstructure and the fracture surfaces of the Al/B-diamond composites were analyzed by SEM and energy dispersive X-ray spectroscopy (EDS). The interfacial structure of the composites was studied by high-resolution transmission electron microscopy (HRTEM, Talos F200X, Waltham, MA, USA) and energy dispersive X-ray spectroscopy (EDS). The TEM samples were prepared using a focused ion beam workstation (FIB, FEI Nova 200, Hillsborough, OR, USA).

The thermal conductivity of the diamond particles was tested by a time-domain thermoreflectance technique, as shown in Table 1. The thermal conductivity of the Al/B-diamond composites was calculated using $K=\alpha \cdot \rho \cdot C_{p}$, where *K* is thermal conductivity, *α* is thermal diffusivity, *ρ* is the density, and *C_p_* is specific heat capacity. For *α* testing, the Al/B-diamond composite compacts were cut into disc-shaped samples of Φ 10 mm × 3 mm. The *α* was measured by laser flash analyzer (LFA 427, Netzsch, Selb, BY, Germany). The *ρ* was determined by Archimedes’ method, and the *C_p_* was examined by differential scanning calorimetry (DSC 204, Netzsch, Selb, BY, Germany). The coefficient of thermal expansion of the Al/B-diamond composites was detected by thermal expansion instrument (DIL402, Netzsch, Selb, BY, Germany) with a heating rate of 5 °C/min from 50 °C to 400 °C. The samples of the coefficient of thermal expansion were processed into a cylindrical shape of Φ 5 mm × 25 mm. Each experimental data point was measured at least three times to obtain the average of the results.

## 3. Results and Discussion

### 3.1. Characterization of B-Coated Diamond Particles

**Figure 2:**
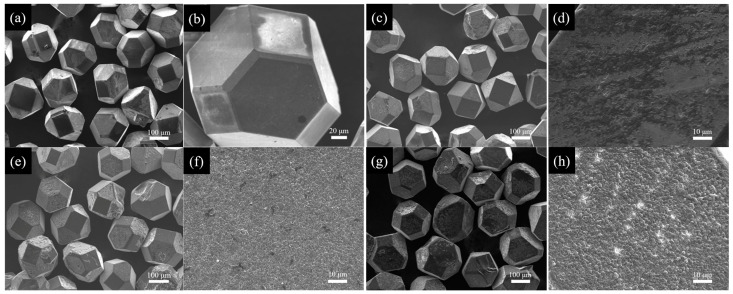
The SEM images of the diamond particles with different plating times: (**a**,**b**) uncoated; (**c**,**d**) 30 min; (**e**,**f**) 90 min; (**g**,**h**) 120 min.

The diamond particles were coated by B at 1250 °C with different plating times. Figure 2 shows the surface morphology of the uncoated and B-coated diamond particles. The diamond particles maintain the regular shape before and after coating with a distinct hexahedral-octahedral structure, consisting of six square {100} faces and eight hexagonal {111} faces. The surface of the uncoated diamond particles is smooth, as shown in Figure 2a,b. On the contrary, Figure 2c–h shows the rough surface of B-coated diamond particles at 30 min, 90 min, and 120 min, respectively. The residuals on the surfaces of the B-coated diamond particles are the products of interfacial reaction in the vacuum thermal diffusion. Figure 2c,d reveals the morphology of the B-coated diamond particles at plating time for 30 min. The {100} faces of the diamond particles are completely and uniformly covered by the coating, while the coating on the {111} faces is discontinuous, exhibiting lamellar or island-like morphologies. This discovery means that the B coating is formed in a vacuum heated at 1250 °C for 30 min. However, when the plating time is extended to 90 min and 120 min, respectively. The surfaces of the diamond particles are completely and uniformly covered by the B coating layer, as shown in Figure 2e–h.

**Figure 3:**
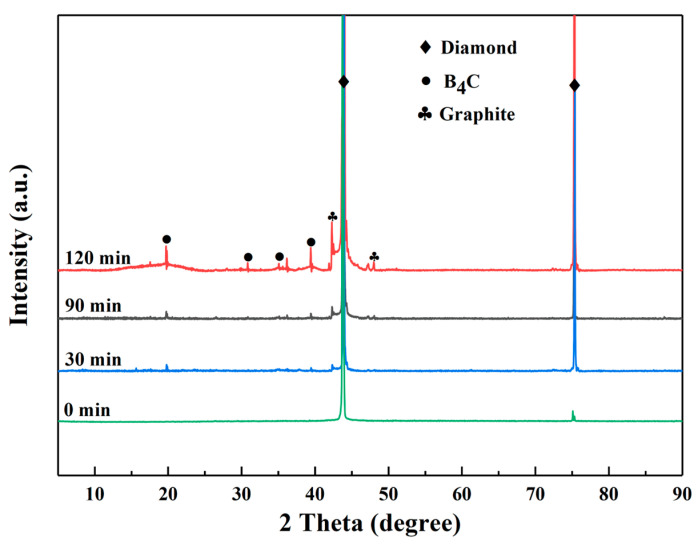
XRD patterns of B-coated diamond particles with different plating times.

Figure 3 shows the XRD patterns of the B-coated diamond particles with various plating times at 1250 °C. The diffraction peaks correspond to diamond (PDF#06-0675), B_4_C (PDF#35-0798), and graphite (PDF#41-1487). The results show that the B coating is composed of the B_4_C phase. The B_4_C was formed by a chemical reaction between plated B atoms and C atoms from diamond particles. The intensity of the diffraction peaks of B_4_C increases with the prolonged plating time. This suggests that the coating thickness gradually increases with prolonging plating time. When the plating time is longer than 90 min, the diffraction peak of graphite is found. This indicates that the C atoms on the surface of diamond particles undergo graphitization transformation at the plating time of 120 min. This is consistent with the results in reference [31].

**Figure 4:**
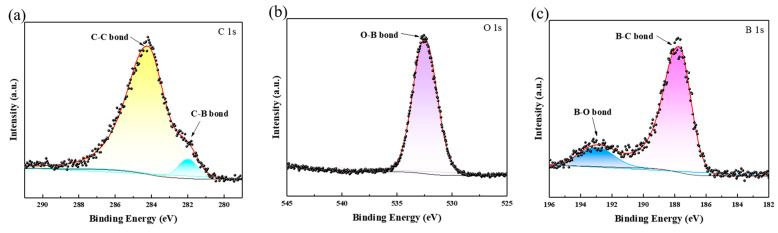
XPS spectra of B-coated diamond particles with 120 min plating time: (**a**) high-resolution spectra of C 1s; (**b**) high-resolution spectra of O 1s; (**c**) high-resolution spectra of B 1s.

The XPS analyses of the B-coated diamond particles were used to investigate the chemical state of the B coating layer. Figure 4 displays the C 1s, O 1s, and B 1s angle-resolved spectra of the B-coated diamond particles with 120 min plating time. The C 1s spectra exhibit two distinct peaks, corresponding to 284.2 eV for the C-C bond and 282 eV for the C-B bond, respectively. One characteristic peak is the O-B bond at 532.4 eV in the O 1s spectra. The B 1s spectra consist of two distinct doubles. The peak at 193.2 eV is attributed to the B-O bond, and 187.7 eV is the B-C bond. The results indicate that the presence of graphitization transformation of the diamond and the chemical reaction between B-plated atoms and C atoms from diamond particles with 120 min plating time. The B coating is mainly composed of the B_4_C phase. The O-B bond is formed by a chemical reaction between the residual O atoms and B-plated atoms at high temperatures.

### 3.2. Microstructure of Al/B-Diamond Composites

**Figure 5:**
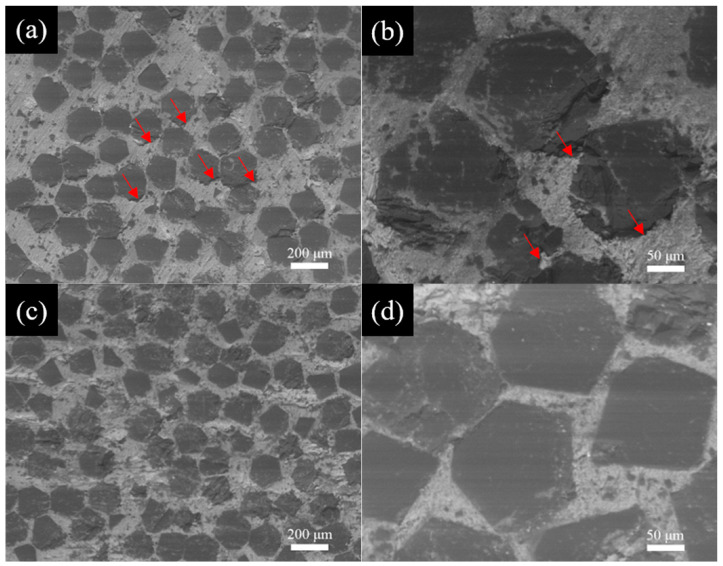
SEM micrographs of Al/B-diamond composites: (**a**,**b**) Al/diamond composite; (**c**,**d**) Al/B-diamond composite with 90 min plating time.

Figure 5 shows the SEM micrographs of Al/B-diamond composites prepared by the gas pressure infiltration method. The black areas represent diamond particles, and the gray is the Al matrix in images of the backscattered electron. The diamond particles are homogeneously dispersed in the Al matrix. The volume fraction of diamond particles is approximately 63% in the composites. Some cracks are observed at the interface between the Al matrix and diamond particles in the Al/diamond composite (as indicated by the red arrows in Figure 5a,b). This is attributed to the poor wettability between the Al matrix and diamond particles, which makes it difficult for the Al liquid to fill the gaps between the diamond particles. On the contrary, no defects such as pores or cracks are observed in the Al/B-diamond composite with a 90 min plating time. This suggests that the B_4_C coating layer improves the wettability between the Al matrix and diamond particles, which is beneficial for improving the interface bonding and properties of the Al/B-diamond composites.

**Figure 6:**
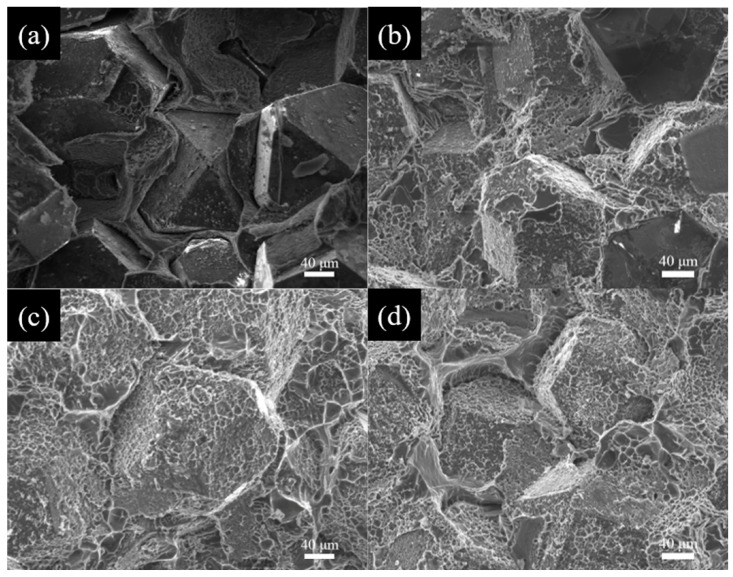
SEM micrographs of the fracture surfaces of Al/B-diamond composites with different coating times at 1250 °C: (**a**) uncoated; (**b**) 30 min; (**c**) 90 min; (**d**) 120 min.

The interfacial bonding mode plays a critical role in the property of composites. In order to further investigate the effect of B_4_C coatings on the interfacial bonding in Al/B-diamond composites. The micrographs of the fracture surfaces of Al/B-diamond composites were systematically analyzed, as shown in Figure 6. The composites displayed obvious changes in the fracture morphologies with different plating times. Figure 6a shows that the uncoated diamond particles almost maintain their original smooth surfaces and are pulled out of the Al matrix. The result suggests that there is a weak mechanical interface bonding between uncoated diamond particles and the Al matrix, and a little of an interfacial reaction occurs on the (100) surface of the diamond in the Al/diamond composite. This is due to the weak wettability between liquid Al and diamond particles, which prevents sufficient contact between the liquid Al and the surface of diamond particles. The fracture mode is that diamond reinforcements are pulled out from the Al matrix.

As compared in Figure 6b, a large number of attachments are observed on the diamond surface. This indicates that B_4_C coating has improved interfacial bonding in the Al/B-diamond composites. However, some diamond particles still maintain their original surfaces. This is because the diamond particles cannot be completely covered by the B coating when the plating time is 30 min, as shown in Figure 2d. The fracture mechanism involves the ductile fracture of the Al matrix covered on the diamond surface and interfacial debonding between diamond particles and the Al matrix.

Figure 6c shows that no exposed diamond surface is observed when the plating time increased to 90 min. The Al matrix is uniformly and completely covered on the surface of diamond particles. The fracture dimple of Al matrix on the diamond particles’ surface is found. This suggests that the interfacial bonding strength is higher than the intrinsic strength of the Al matrix, resulting in residual Al on diamond surfaces after the Al/B-diamond composites fracture. The complete B_4_C coating was formed on diamond surfaces at the plating time of 90 min in comparison with 30 min. The result displays that the complete B coating improves the interfacial bonding between the Al matrix and diamond reinforcements. In this case, the fracture mechanism of the Al/B-diamond is dominated by ductile fracture of the Al matrix.

The interfacial debonding is observed again when the plating time is extended to 120 min, as shown in Figure 6d. The peeled interface can be clearly visible between the Al matrix and B-coated diamond particles. This implies that the interface bonding strength is decreased. Figure 3 shows that the XRD analysis displays that the B_4_C coating gradually thickens with the prolongation of plating time. The B_4_C is a brittle phase and could result in low interface bonding strength between the Al matrix and B-coated diamond particles. The fracture mechanism is also a combination of ductile fracture of the Al matrix covered on diamond and interfacial debonding between the Al matrix and diamond particles. The results show that B_4_C coating can effectively improve the interfacial bonding between the Al matrix and diamond particles if the B_4_C coating has appropriate thickness.

**Figure 7:**
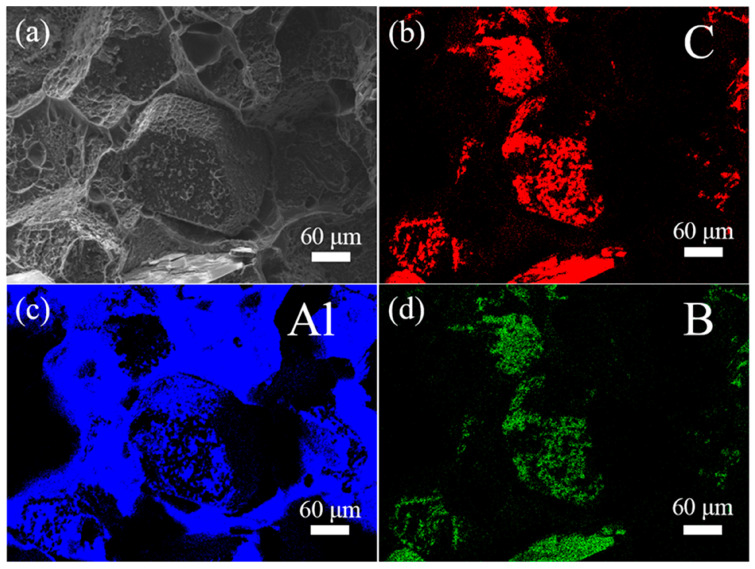
EDS element distribution mappings on the fracture surface of the Al/B-diamond composite with 120 min plating time: (**a**) secondary electron image; (**b**) C element; (**c**) Al element; (**d**) B element.

Figure 7 shows the EDS element distribution mappings on the fracture surface of the Al/B-diamond composite with 120 min plating time. Figure 7a is the secondary electron image showing the morphology of the detected area. Figure 7b–d corresponds to the distribution maps of C, Al, and B elements, respectively. It can be clearly observed that Al and B elements are distributed on the entire fracture surfaces of the Al/B-diamond composites. The results confirm that the B_4_C coating has strong interfacial bonding between diamond particles and the Al matrix. On the one hand, it is beneficial for the B coating to completely cover diamond particles during the preparation process of Al/B-diamond composites, avoiding the formation of Al_4_C_3_ by the reaction between diamond particles and Al liquid. On the other hand, it enhances the interfacial bonding of the composites, helping to improve the property of the Al/B-diamond composites. Therefore, the B_4_C coating plays a good transitional role at the interface of the Al/B-diamond composites.

**Figure 8:**
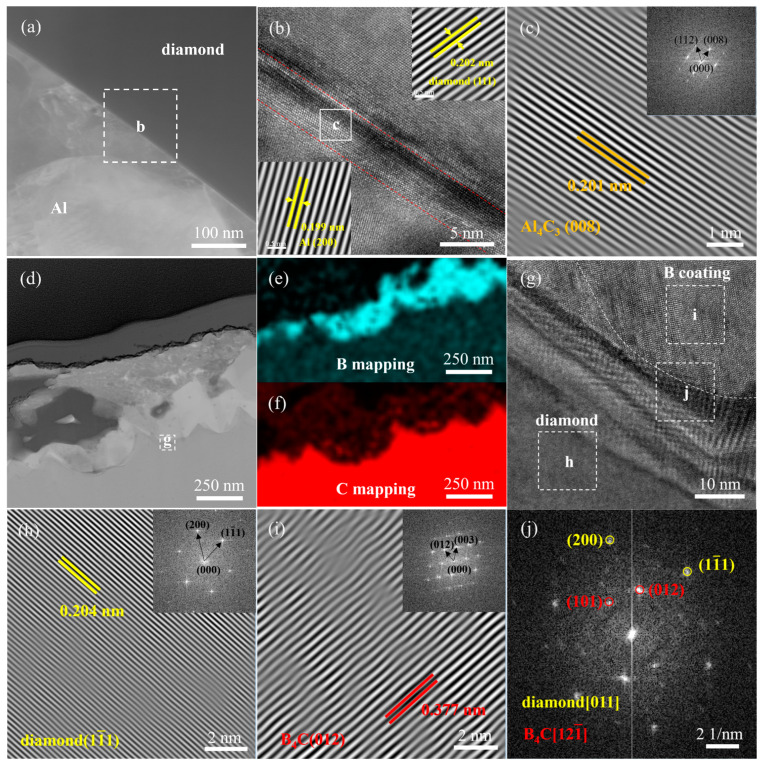
TEM analysis of the interfacial structure of the Al/B-diamond composites: (**a**) uncoated; (**b**) HRTEM image of region b in (**a**); (**c**) IFFT image of region c in (**b**); (**d**) 90 min plating time; (**e**,**f**) EELS elemental maps of (**d**); (**g**) HRTEM image of region g in (**d**); (**h**,**i**) IFFT images of region h and region i in (**g**); (**j**) SAED patterns of region j in (**g**).

To further investigate the influence of B_4_C coating on the interface of Al/B-diamond composites, the interfacial microstructure of the Al/B-diamond composites was characterized by TEM, as shown in Figure 8. During the preparation process of Al/diamond composites, the interfacial layer of Al_4_C_3_ with a thickness of approximately 5 nm was formed along the diamond boundary, as shown in Figure 8a–c. On the contrary, Figure 8d–j shows the interfacial microstructure of Al/B-diamond composites with 90 min plating time. Figure 8e shows the average thickness of the B coating is about 250 nm. The interface layer is only composed of B_4_C, and Al_4_C_3_ is not detected, as shown in Figure 8i,j. The crystallographic orientation relationship between diamond particles and the B_4_C coating is determined to be [011]_diamond_//[1–12]_B4C_ based on the selected area electron diffraction (SAED) pattern shown in Figure 8j. The results indicate that the B_4_C coating on the diamond surface is stable and maintains its composition during the infiltration of molten Al, which avoids the formation of the interfacial reaction product Al_4_C_3_.

### 3.3. Thermal-Physical Properties of Al/B-Diamond Composites

**Figure 9:**
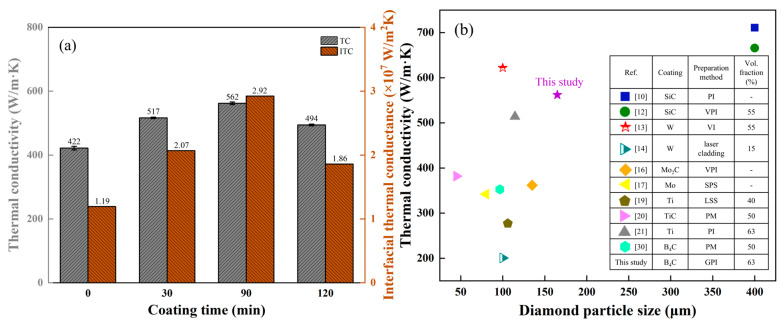
(**a**) The thermal conductivity and interfacial thermal conductance of Al/B-diamond composites; (**b**) comparison of thermal conductivity with literature.

Figure 9 shows the thermal conductivity of Al/B-diamond composites with different plating times. The Al/diamond composite shows the lowest thermal conductivity of 422 W/m·K. This is attributed to the interface defect in the Al/diamond composite (Figure 5a) and the weak interfacial bonding between uncoated diamond particles and the Al matrix (Figure 6a). By contrast, the Al/B-diamond composites display the higher thermal conductivity. With the coating time increasing from 30 min to 120 min, the thermal conductivity of Al/B-diamond composites first increases and then decreases. At the plating time of 90 min, the thermal conductivity of the Al/B-diamond composite exhibits a maximum value of 562 W/m·K, increasing by 33% compared to the Al/diamond composite. The results are due to interfacial modification between the Al matrix and B-coated diamond particles. When the plating time is 30 min, the B coating on the (100)_diamond_ exhibits uniformity, but the (111)_diamond_ is not complete, as shown in Figure 2d, which has limited improvement in the interfacial bonding of Al/B-diamond (Figure 6b). The SEM analyses for B-coated diamond particles and TEM analyses for Al/B-diamond composite with a plating time of 90 min show that the complete B coating consists of a B_4_C layer with a thickness of ~250 nm, as shown in Figure 2f and Figure 8e. The B_4_C layer with such an appropriate thickness provides positive interfacial bonding between the Al matrix and diamond particles.

It is worth noting that the thermal conductivity decreased at the plating time of 120 min. The result is attributed to the high interfacial thermal resistance by the low thermal conductivity of B coating. Meanwhile, the graphitization transformation of carbon atoms on the surface of diamond will occur at the plating time of 120 min, as shown in Figure 3, which decreased the thermal conductivity of diamond.

In this study, the thermal conductivity of Al/B-diamond composite is significantly higher than previously obtained Al/(W, Mo, Ti, B)-diamond composites [14,16,17,19,20,21,30], as shown in Figure 9b. The result indicates that the B coatings have significant advantages over certain elements in improving the thermal conductivity of Al/diamond composites. As well know the thermal conductivity of the Al/diamond composites increases with increasing the size and volume fraction of diamond particles. Hence, the thermal conductivity of Al/B-diamond composite is lower than that of Al/SiC-diamond composites [10,12]. Overall, the thermal conductivity of Al/diamond composites has been enhanced by B coating on the surface of diamond particles.

The interfacial structure is crucial to the thermal-physical properties of Al/diamond composites. In order to understand the effect of B_4_C coating on the thermal conductivity of Al/B-diamond composites. The interfacial thermal conductance of the composites is calculated by the differential effective medium (DEM) model: [32,33]

$$\left(1-V_{d}\right){\left(\frac{K_{c}}{K_{m}}\right)}^{\frac{1}{3}}=\frac{\frac{K_{d}}{K_{m}\left(1+\frac{K_{d}}{ha}\right)}-\frac{K_{c}}{K_{m}}}{\frac{K_{d}}{K_{m}\left(1+\frac{K_{d}}{ha}\right)}-1}$$

where *K* is thermal conductivity, *h* is the interfacial thermal conductance, *V* is the volume fraction of reinforcement, and *a* is the reinforcement radius. The subscripts *d*, *m*, and *c* are the reinforcement, matrix, and composite, respectively. In this work, the diamond reinforcements are assumed to be isotropic spherical particles. The average radius and volume fraction of diamond in the Al/B-diamond composite are 82.5 μm and 63%, respectively. The thermal conductivities of the Al matrix and diamond are 230 W/m·K and 1415 W/m·K, respectively.

The calculation results of *h* are plotted in Figure 9. The change in tendency of *h* is similar to the thermal conductivity. The *h* of the Al/diamond composites with B-coated diamond particles is obviously higher than the 1.19 × 10^7^ W/m^2^·K of uncoated composites. The *h* highest value of 2.92 × 10^7^ W/m^2^·K for the Al/B-diamond composite at the plating time of 90 min, which is 145% higher than the Al/diamond composite. The results indicate that the continuous B_4_C coating can effectively improve the interfacial thermal conductance of Al/B-diamond composites. Meanwhile, the Debye temperature of B_4_C is about 1480 Θ/K, which is between 428 Θ/K of Al and 2230 Θ/K of diamond [25,26]. This helps to reduce the phonon mismatch between Al and diamond particles. Hence, the B_4_C coating improves the interfacial thermal conductance, resulting in the higher thermal conductivity of Al/B-diamond composites.

**Figure 10:**
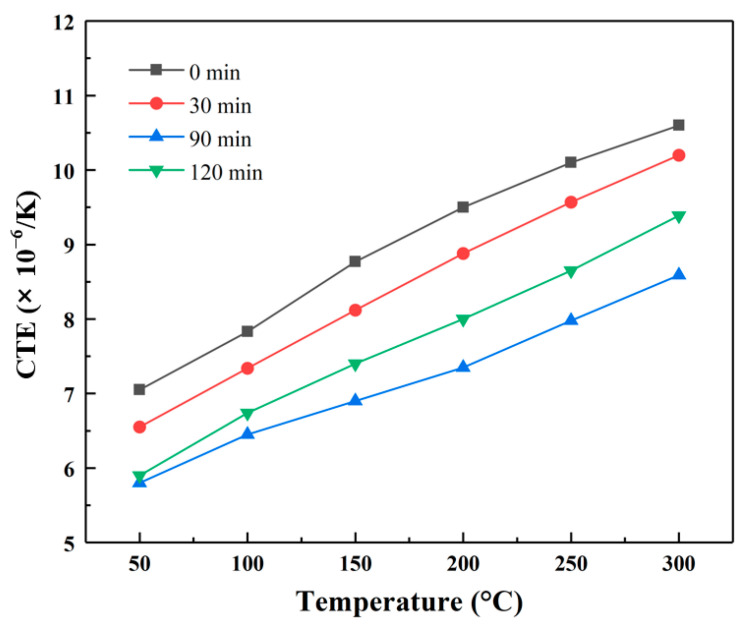
Variation of the coefficient of thermal expansion with temperature for the Al/B-diamond composites.

Figure 10 shows the variation of the coefficient of thermal expansion (CTE) with temperature for the Al/B-diamond composites. The CTE of the Al/B-diamond composites was significantly lower than the CTE of the Al/diamond composite. In the temperature range from 50 °C to 300 °C, the uncoated diamond composite had higher CTEs ranging from 7.0 × 10^−6^/K to 10.6 × 10^−6^/K compared with 5.8 × 10^−6^/K –8.5× 10^−6^/K for the Al/B-diamond composite at the plating time of 90 min. The CTE is decreased 17% and 20%, respectively. The lowered CTEs of the Al/B-diamond composites should be attributed to the positive interfacial bonding between B-coated diamond particles and the Al matrix. The change of CTE with the plating time for the Al/B-diamond composites is evident. It is noted that the CTEs of the Al/B-diamond composite at the plating time of 90 min increase slowly with temperature compared to other plating times. This indicates that, with strong interfacial bonding, the B_4_C coating with low CTE of 5.73 × 10^−6^/K [34] has significantly restricted the expansion of the surrounding Al matrix in the Al/B-diamond composite.

## 4. Conclusions

The B-coated diamond-reinforced Al matrix composites were successfully produced by the gas pressure infiltration method. The influence of B coating on the interface and thermal physical properties of the Al/B-diamond composites was systematically studied. The results show that the B_4_C coating with suitable thickness can effectively improve the thermal physical properties of the Al/B-diamond composites. The main conclusion can be summarized as follows:

(1) The continuous B4C coating layer on the surface of diamond particles was prepared by the vacuum thermal diffusion method at 1250 °C. The B4C coating avoided the formation of Al4C3 at the interface of Al/B-diamond composites.
(2) The B_4_C coating improved the interfacial bonding and interfacial thermal conductance between the Al matrix and diamond particles. The highest interfacial thermal conductance value of 2.92 × 10^7^ W/m^2^K was obtained for the Al/B-diamond composite, increasing 145% compared with the Al/diamond composite.
(3) The thermal conductivity increased from 422 W/m·K for Al/diamond composite to 562 W/m·K for Al/B-diamond composite with 90 min plating time. When the plating time increased to 120 min, the low thermal conductivity was attributed to the thickening of the B_4_C coating with low thermal conductivity and the graphitization of carbon atoms on the surface of the diamond.
(4) The coefficient of thermal expansion of the Al/B-diamond composites was reduced and reached a level of 5.8 × 10^−6^/K–8.5× 10^−6^/K in the temperature range from 50 °C to 300 °C. The strong interfacial bonding between B-coated diamond particles and Al matrix restricted the expansion of the surrounding Al matrix.

## Figures and Tables

**Table 1:** Parameters of the materials.

Materials	Density (g/cm^3^)	TC (W/m·K)	CTE (×10^−6^/K)
Diamond	3.52	1415.0	1.0
Al	2.70	230.0	23.0
B	2.34	27.4	5.7
B_4_C	2.52	67.0	4.5

## Data Availability

All data are available from the corresponding author on reasonable request.

## References

[1] He Z., Yan Y., Zhang Z. (2021). Thermal management and temperature uniformity enhancement of electronic devices by micro heat sinks: A review. Energy.

[2] Molina J.M., Narciso J., Weber L., Mortensen A., Louis E. (2008). Thermal conductivity of Al–SiC composites with monomodal and bimodal particle size distribution. Mater. Sci. Eng. A.

[3] Moore A.L., Shi L. (2014). Emerging challenges and materials for thermal management of electronics. Mater. Today.

[4] Zhu P., Zhang Q., Qu S., Wang Z., Gou H., Shil’ko S.V., Kobayashi E., Wu G. (2022). Effect of interface structure on thermal conductivity and stability of diamond/aluminum composites. Compos. Part A Appl. Sci. Manuf..

[5] Zhang L., Wei Q., An J., Ma L., Zhou K., Ye W., Yu Z., Gan X., Lin C.-T., Luo J. (2020). Construction of 3D interconnected diamond networks in Al-matrix composite for high-efficiency thermal management. Chem. Eng. J..

[6] Yang W., Chen G., Wang P., Qiao J., Hu F., Liu S., Zhang Q., Hussain M., Dong R., Wu G. (2017). Enhanced thermal conductivity in Diamond/Aluminum composites with tungsten coatings on diamond particles prepared by magnetron sputtering method. J. Alloys Compd..

[7] Che Z., Zhang Y., Li J., Zhang H., Wang X., Sun C., Wang J., Kim M.J. (2016). Nucleation and growth mechanisms of interfacial Al_4_C_3_ in Al/diamond composites. J. Alloys Compd..

[8] Rodríguez-Reyes M., Pech-Canul M.I., Parga-Torres J.R., Acevedo-Dávila J.L., Sánchez-Araiza M., Lopez H.F. (2011). Development of aluminum hydroxides in Al–Mg–Si/SiC_p_ in infiltrated composites exposed to moist air. Ceram. Int..

[9] Lu Y., Wang X., Zhang Y., Wang J., Kim M.J., Zhang H. (2018). Aluminum carbide hydrolysis induced degradation of thermal conductivity and tensile strength in diamond/aluminum composite. J. Compos. Mater..

[10] Sang J., Chen Q., Yang W., Zhu J., Fu L., Li D., Zhou L. (2022). Architecting micron SiC particles on diamond surface to improve thermal conductivity and stability of Al/diamond composites. Surf. Interfaces.

[11] Liu P., He X., Qu X. (2022). Effect of diamond surface structure on the interfacial reaction and properties of diamond/SiC composites. Diamond Relat. Mater..

[12] Li X., Yang W., Sang J., Zhu J., Fu L., Li D., Zhou L. (2020). Low-temperature synthesizing SiC on diamond surface and its improving effects on thermal conductivity and stability of diamond/Al composites. J. Alloys Compd..

[13] Xin L., Tian X., Yang W., Chen G., Qiao J., Hu F., Zhang Q., Wu G. (2018). Enhanced stability of the Diamond/Al composites by W coatings prepared by the magnetron sputtering method. J. Alloys Compd..

[14] Huang S., Zhao Y., Xie H., Guo H., Peng L., Zhang W. (2024). Microstructure and Properties of Aluminum Alloy/Diamond Composite Materials Prepared by Laser Cladding. Materials.

[15] Chen G., Yang W., Xin L., Wang P., Liu S., Qiao J., Hu F., Zhang Q., Wu G. (2018). Mechanical properties of Al matrix composite reinforced with diamond particles with W coatings prepared by the magnetron sputtering method. J. Alloys Compd..

[16] Ma S., Zhao N., Shi C., Liu E., He C., He F., Ma L. (2017). Mo_2_C coating on diamond: Different effects on thermal conductivity of diamond/Al and diamond/Cu composites. Appl. Surf. Sci..

[17] Lu Y., Chen W., Wang C., Tian W., He J., Liao W. (2024). Influence mechanism of magnetron sputtering process parameters on diamond surface preprocessing interface for chip heat sink. Diam. Relat. Mater..

[18] Li K., Hu Z., Yang W., Duan W., Ni X., Hu Z., He W., Cai Z., Liu Y., Zhao Z. (2024). Selective laser melting and mechanical behavior of Mo-coated diamond particle reinforced metal matrix composites. Diam. Relat. Mater..

[19] Zhou H., Jia Q., Sun J., Li Y., He Y., Bi W., Zheng W. (2024). Improved Bending Strength and Thermal Conductivity of Diamond/Al Composites with Ti Coating Fabricated by Liquid-Solid Separation Method. Materials.

[20] Liu X.Y., Wang W.G., Wang D., Ni D.R., Chen L.Q., Ma Z.Y. (2016). Effect of nanometer TiC coated diamond on the strength and thermal conductivity of diamond/Al composites. Mater. Chem. Phys..

[21] Guo C.Y., He X.B., Ren S.B., Qu X.H. (2016). Thermal properties of diamond/Al composites by pressure infiltration: Comparison between methods of coating Ti onto diamond surfaces and adding Si into Al matrix. Rare Metals.

[22] Krstic Z., Mecca W., Haerle A.G., Tumavitch N.J., Shaffer B.C. (2015). Nickel Coated Diamond Particles and Method of Making Said Particles. U.S. Patent.

[23] Mudholkar M.S., Goetz R.J. (2004). Autocatalytic Nickel-Boron Coating Process for Diamond Particles. U.S. Patent.

[24] Ras A.H., Auret F.D., Nel J.M. (2010). Boron carbide coatings on diamond particles. Diam. Relat. Mater..

[25] Hua Z., Wang K., Li W., Chen Z. (2023). Theoretical Strategy for Interface Design and Thermal Performance Prediction in Diamond/Aluminum Composite Based on Scattering-Mediated Acoustic Mismatch Model. Materials.

[26] Yuan M., Tan Z., Fan G., Xiong D.-B., Guo Q., Guo C., Li Z., Zhang D. (2018). Theoretical modelling for interface design and thermal conductivity prediction in diamond/Cu composites. Diam. Relat. Mater..

[27] Bai G., Li N., Wang X., Wang J., Kim M.J., Zhang H. (2018). High thermal conductivity of Cu-B/diamond composites prepared by gas pressure infiltration. J. Alloys Compd..

[28] Hu H., Kong J. (2013). Improved Thermal Performance of Diamond-Copper Composites with Boron Carbide Coating. J. Mater. Eng. Perform..

[29] Guo R.-F., Chen S.-M., Shen P. (2023). Influence of Si, Ti, and Cu as alloying elements on the wettability and reactivity of an Al/B_4_C system. J. Mater. Res. Technol..

[30] Sun Y., Zhang C., He L., Meng Q., Liu B.C., Gao K., Wu J. (2018). Enhanced bending strength and thermal conductivity in diamond/Al composites with B_4_C coating. Sci. Rep..

[31] Yan X., Wei J., An K., Liu J., Chen L., Zheng Y., Zhang X., Li C. (2021). High temperature surface graphitization of CVD diamond films and analysis of the kinetics mechanism. Diam. Relat. Mater..

[32] Every A.G., Tzou Y., Hasselman D.P.H., Raj R. (1992). The effect of particle size on the thermal conductivity of ZnS/diamond composites. Acta Metall. Mater..

[33] Bruggeman D.A.G. (1935). Berechnung verschiedener physikalischer Konstanten von heterogenen Substanzen. I. Dielektrizitätskonstanten und Leitfähigkeiten der Mischkörper aus isotropen Substanzen. Ann. Phys..

[34] Tsagareishvili G.V., Nakashidze T.G., Jobava J.S., Lomidze G.P., Khulelidze D.E., Tsagareishvili D.S., Tsagareishvili O.A. (1986). Thermal expansion of boron and boron carbide. J. Less Common. Met..

